# Review of "bioinformatics basics: applications in biological science and medicine" by Buehler & Rashidi

**DOI:** 10.1186/1475-925X-5-41

**Published:** 2006-06-19

**Authors:** Aedín Culhane

**Affiliations:** 1 Dana-Farber Cancer Institute and Harvard School of Public Health, Department of Biostatistics and Computational Biology, 44 Binney Street, Boston, Massachusetts 02115, USA

I was excited to receive the second edition of this book for review. The first edition published in 2000 received good reviews on Amazon (four stars based on 5 reviews). Hence I had expected a book which provided a broad introduction to bioinformatics as applied to biology and medicine; however I regret to say I was disappointed.

This book is divided into 5 chapters. The first chapter 'Biology and Information' begins with an interesting but lengthy and somewhat unfocused introduction to the application of computers in biology and medicine. This is followed by a useful and concise section in basic DNA and protein biochemistry, structure and function.

Databases are central to bioinformatics and chapter 2 on 'Biological Databases' should have provided a useful resource to novices to the field, however this chapter has been haphazardly updated from the first edition. Many details in this chapter are out of date. For example, we are told that "the basic BLAST program does not allow gaps in its alignment" and that the executables for the "new" version of BLAST are available for "IRIX 6.2, Solaris 2.5, DEC OSF1 and Win 32 operating systems." This was correct in 1997 (BLAST version 2.0), but is no longer correct and should have been updated. In other instances statistics on the number of entries in a database are given, however a date or release version is not provided, thus making this information meaningless. This chapter is structured around a description of the web resources provide by the large bioinformatic institutes (NCBI, EBI). This results in a chapter with poor layout and repetition. For example, interpretation of the BLAST E-value statistic is described twice. This layout is unsatisfactory also as it tends to focus on older databases ignoring valuable recent resources. It is a pity that the authors did not direct the reader to the annual Nucleic Acids Research Database and Web Server issues. Major themes central to Bioinformatics such as multiple alignment and phylogenetic tree analysis are poorly addressed. This section briefly mentions ClustalW and directs the reader to the NCBI databases of orthologous genes, Homologene and PFAM. With increasing numbers of complete genomes available, a more comprehensive introduction to phylogenetic analysis and comparative genomics would be valuable.

A highlight of the book is the well written and informative section on microarrays which was contributed by Thorsten Forster and Peter Ghazal in Chapter 3. Unfortunately this did not include examples of applications of microarrays to biological science or medicine. Chapter 4 which focuses on proteome analysis contained sections on 2D gels, protein-protein interaction data, protein structure (PDB) and homology modeling of protein structure. The latter focused almost exclusively commercial software from Accelrys. A large proportion of this chapter (30 pages) was devoted to a detailed description of analytical ultracentrifugation and hydrodynamics. Although this was interesting, the depth of this section may have beyond the scope of this book. Chapter 5 which promised to provide an overview of applications of bioinformatics was brief (19 pages) and general.

As this book is entitled Bioinformatics Basics: Applications in Biological Science and Medicine, I expected this book to provide a solid foundation in basic bioinformatics, together with interesting case studies where bioinformatics has been applied. Whilst this book may have filled in niche in 2000 when few books were available, numerous publications which provide good introductions to bioinformatics are now available. This book provides an easy introduction but the careless editing of the second version of this book maybe distracting to the readership of Biomedical Engineering Online.

